# 25-Gauge Microincision Vitrectomy to Treat Vitreoretinal Disease in Glaucomatous Eyes after Trabeculectomy

**DOI:** 10.1155/2014/306814

**Published:** 2014-04-22

**Authors:** Hiroshi Kunikata, Naoko Aizawa, Nobuo Fuse, Toshiaki Abe, Toru Nakazawa

**Affiliations:** ^1^Department of Ophthalmology, Tohoku University Graduate School of Medicine, 1-1 Seiryo-machi, Aoba-ku, Sendai 980-8574, Japan; ^2^Department of Integrative Genomics, Tohoku Medical Megabank Organization, Tohoku University Graduate School of Medicine, Sendai 980-8573, Japan; ^3^Division of Clinical Cell Therapy, Tohoku University Graduate School of Medicine, Sendai 980-8575, Japan

## Abstract

*Purpose*. To determine the feasibility of using 25-gauge microincision vitrectomy surgery (25GMIVS) to treat vitreoretinal disease in glaucomatous eyes which have previously undergone trabeculectomy (TLE). *Methods*. A consecutive, interventional case series. We performed 25GMIVS in 15 glaucomatous eyes that had undergone TLE. Follow-up period was 11.5 months. *Results*. 25GMIVS was successfully used and led to improvement in visual acuity (*P* < 0.01). We performed 25GMIVS for proliferative diabetic retinopathy with neovascular glaucoma in 53% of eyes (8 of 15). Although 3 eyes needed further TLE following 25GMIVS, final IOP was below 21 mmHg in all eyes except one eye (93%) and was comparable to pre-25GMIVS IOP (*P* = 0.20) without an increase in the number of glaucoma medications (*P* = 0.14). *Conclusions*. 25GMIVS is a feasible treatment for vitreoretinal disease in eyes with preexisting TLE, effective in both significantly improving BCVA and preserving the filtering bleb, while not excluding further glaucoma surgery.

## 1. Introduction

Trabeculectomy (TLE) is a procedure most often performed when drug-based therapies for glaucoma have been ineffective. It can effectively reduce intraocular pressure (IOP) over the long term [1–3], but can lead to problems if further severe retinal diseases requiring vitrectomy arise. This is particularly the case for conventional 20-gauge par planar vitrectomy (20GPPV), because that procedure requires suturing and a conjunctival incision, which can disrupt the ocular surface and lead to impairment of the filtering bleb [4]. Furthermore, 20GPPV can make future or unanticipated filtering surgery more difficult as it causes conjunctival-scleral adhesion in multiple quadrants. It would thus be desirable to establish an alternative vitrectomy technique that has a lower risk of causing filtering bleb failure and does not exclude further glaucoma surgery.

Twenty-five-gauge microincision vitrectomy surgery (25GMIVS) was first reported in 2002, and this procedure is now commonly used worldwide [5–15]. One of the advantages of this technique is that intraoperative suturing is not needed, which reduces postoperative ocular pain and discomfort in patients. Furthermore, 25GMIVS allows earlier postoperative visual improvement than 20GPPV and does not induce significant changes in the corneal topography or optical quality of the cornea [16–20]. Although 25GMIVS does have limitations [21–23], we believe that because it is sutureless, it is the best choice to treat retinal disease in eyes with preexisting TLE and can best preserve the filtering bleb. However, to the best of our knowledge, using the PubMed search system, there are no reports discussing or evaluating the use of 25GMIVS to treat retinal diseases in glaucomatous eyes after TLE, except one case report of familial amyloid polyneuropathy (FAP) [24].

Thus, the purpose of this study was to determine the feasibility of using 25GMIVS to treat vitreoretinal disease in glaucomatous eyes that have undergone TLE.

## 2. Patients and Methods

### 2.1. Participants

This was a retrospective, consecutive, interventional case series performed at a single center. Fifteen consecutive post-TLE eyes of 15 patients with retinal diseases who underwent 25GMIVS were studied. The inclusion criterion was any retinal vitreous disease causing visual dysfunction in eyes that had previously undergone TLE, (meaning the eyes already had a filtering bleb). The exclusion criteria were prior scleral buckling, prior trauma, and a follow-up period of less than 3 months. The preoperative demographics and postoperative courses of the patients are shown in Table 1. All of the surgeries were performed at the Surgical Retina Service of Tohoku University Hospital from October 2008 to May 2012. All 25GMIVS procedures were performed by a single surgeon (H.K.). After the purpose and procedures of the operation were explained, informed consent was obtained from all patients. This study conformed to the tenets of the Declaration of Helsinki and was approved by the Institutional Review Board of the School of Medicine, Tohoku University.

### 2.2. Surgical Procedures

All surgeries were performed under retrobulbar anesthesia using the oblique sclerotomy technique and were performed using the Accurus Vitrectomy System (Alcon Laboratories; Fort Worth, Texas, USA). First, an infusion cannula was inserted through the inferotemporal sclera followed by the insertion of two cannulas through superotemporal and superonasal sites. The insertion point of the cannulas was shifted as necessary to avoid disturbing the conjunctiva adjacent to the filtering bleb. Next, a 2.4 mm superotemporal corneal incision was made, followed by phacoemulsification, aspiration (PEA), and intraocular lens (IOL) implantation before the vitrectomy, if the eye had a cataract. After resecting the vitreal core, 4 mg of triamcinolone acetonide (TA; Kenacort-A, Bristol-Meyers Squibb, Tokyo, Japan) was injected into the vitreous cavity to determine if a posterior vitreous detachment (PVD) was present. If a PVD was not present, we created one with a 25-gauge cutter. After shaving the peripheral gel, the proliferative membrane was removed, and fluid air exchange and endophotocoagulation were performed if needed. The exact surgical procedures varied according to the type of vitreoretinal disease. Additional TLE was also performed under retrobulbar anesthesia, with a fornix-based conjunctival flap. A half-thickness 4.0 by 4.0 mm rectangular scleral flap was made in the superior area. Mitomycin C (MMC) was used with a concentration of 0.04% and an exposure time of 5 minutes. The area was irrigated thoroughly with 200 mL of balanced salt solution. Trabeculectomy was then performed, followed by peripheral iridectomy. The scleral flap and conjunctiva were closed with a 10-0 nylon suture. Postoperatively, antibiotics and corticosteroids were injected subconjunctivally.

### 2.3. Measurements of Clinical Findings

We evaluated best-corrected visual acuity (BCVA), IOP, number of glaucoma medications, intraoperative subconjunctival hemorrhage, intraoperative suturing at the sclerotomy site, and additional TLE (after 25GMIVS). BCVA was measured using the Landolt C visual acuity chart, and the decimal BCVA was converted to logarithm of the minimal angle of resolution (LogMAR) units for statistical analysis. Success of the 25GMIVS procedure was defined as improvement or maintenance of BCVA and maintenance of IOP ≤21 mmHg with the use of topical glaucoma medication, with no need for additional TLE. The procedure was recorded as a failure if there was a decrease in BCVA, additional TLE was required, or IOP could not be maintained ≤21 mmHg with the use of topical glaucoma medication.

### 2.4. Statistical Analysis

The data are presented as the mean ± standard deviation. The significance of the difference between the pre-25GMIVS BCVA and final BCVA in logMAR units was determined by the single tailed paired* t*-test. For the statistical analysis, “count fingers” visual acuity was set as 2.0 logMAR units, and “hand motion” acuity was set as 3.0 logMAR units. The significance of the difference in IOP before TLE, after TLE, and after 25GMIVS was determined by the Friedman test, and the significance of the difference between the IOP after TLE and after 25GMIVS was determined by the Scheffe's test. The significance of the difference in the pre-25GMIVS number of glaucoma medications and the final number was also determined by the two tailed paired* t*-test. A* P* value of less than 0.05 was considered to be statistically significant.

## 3. Results

**Figure 1:**
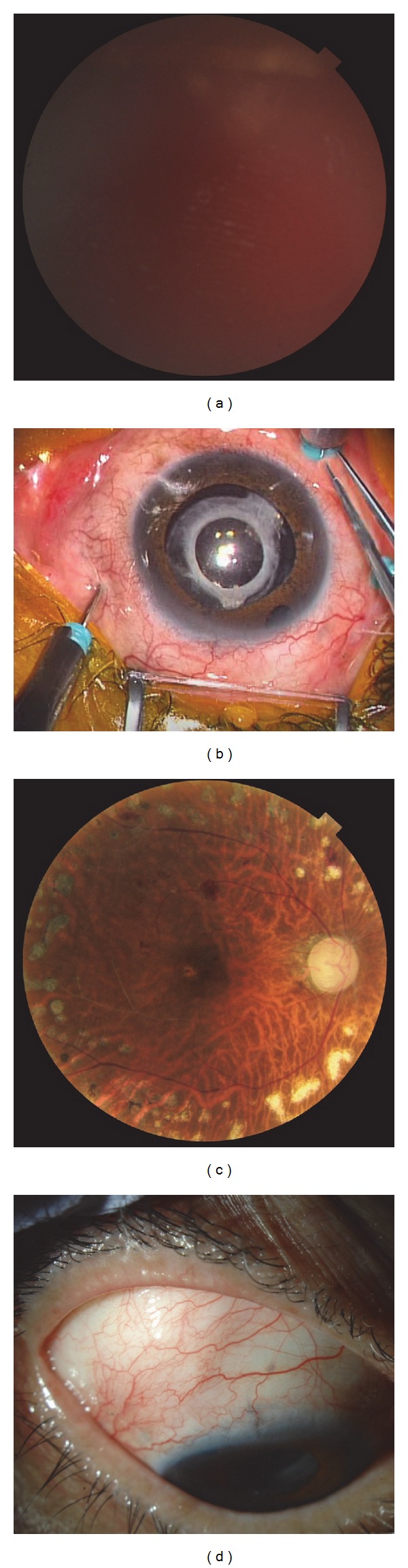
Representative example of proliferative diabetic retinopathy (PDR) complicated by neovascular glaucoma (NVG) (Patient 12; see Table 1). Fundus, anterior segment, and intraoperative photographs of the eye of a 61-year-old man with PDR/NVG. The eye underwent 25-gauge microincision vitrectomy surgery (25GMIVS) after trabeculectomy. (a) Preoperative photograph of the fundus. We could not visualize the posterior fundus due to vitreous hemorrhage (VH). (b) Intraoperative photograph of the anterior segment. 25GMIVS was being performed with 3 ports. The insertion placement of the cannulas was shifted to avoid disturbing the subconjunctival hemorrhage of the filtering bleb in the upper temporal region. (c) Postoperative photograph of the fundus. The VH has been removed and the retinal surface can be seen clearly. (d) One-day postoperative photograph of the anterior segment. There was no subconjunctival hemorrhage, including the filtering bleb, in the upper temporal region.

**Figure 2:**
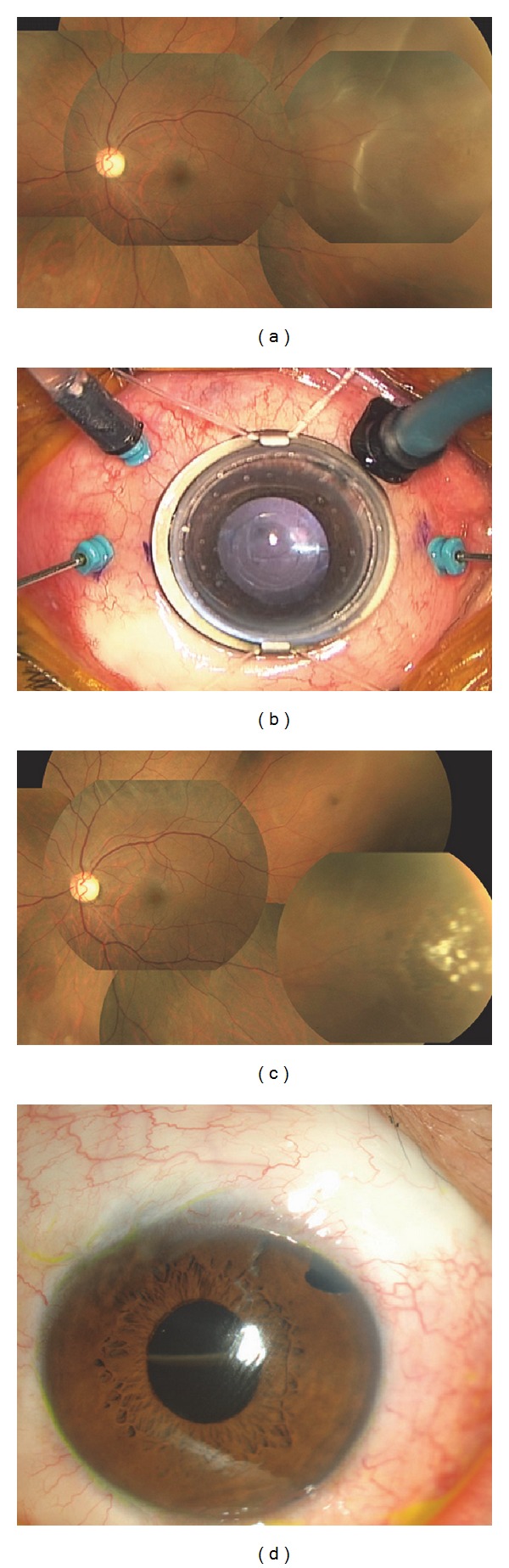
Representative example of rhegmatogenous retinal detachment (RRD) (Patient 1; see Table 1). Fundus, anterior segment, and intraoperative photographs of the eye of a 45-year-old man with RRD. The eye underwent 25-gauge microincision vitrectomy surgery (25GMIVS) after trabeculectomy. (a) Preoperative photograph of the fundus. There was focal retinal detachment with a peripheral retinal tear. (b) Intraoperative photograph of the anterior segment. 25GMIVS was being performed with 4 ports. The insertion placement of the cannulas was shifted to avoid disturbing the subconjunctival hemorrhage of the filtering bleb in the upper temporal region. (c) Postoperative photograph of the fundus. Retinal reattachment was achieved with 25GMIVS. The white retinal scars of endophotocoagulation can be seen. (d) One-day postoperative photograph of the anterior segment. There was no subconjunctival hemorrhage, including the filtering bleb, in the upper temporal region. An air-fluid level line of intraocular gas tamponade can be seen through the pupil.

A summary of the patients' characteristics and pre-25GMIVS course is shown in Table 1. There were 8 men and 7 women with a mean age of 57.4 ± 13.2 years. The type of glaucoma originally requiring TLE included neovascular glaucoma (NVG, 8 eyes; 53%), open angle glaucoma (2 eyes; 13%), malignant glaucoma (2 eyes; 13%), traumatic glaucoma (1 eye), uveitis-associated secondary glaucoma (1 eye), and developmental glaucoma (1 eye). Vitreoretinal diseases in eyes with preexisting-TLE treated with 25GMIVS in our study included proliferative diabetic retinopathy (PDR, 7 eyes; 47%) (Figure 1), malignant glaucoma (2 eyes; 13%), rhegmatogenous retinal detachment (1 eye) (Figure 2), branch retinal vein occlusion (1 eye), macular hole (1 eye), dislocated intraocular lens (1 eye), endophthalmitis (1 eye), and choroidal hemorrhage (1 eye). The mean period between the original TLE procedure and 25GMIVS was 25.3 ± 29.7 months, with a range of 0.3 to 105 months. All blebs were located in the upper quadrants. Prior to TLE, vitrectomy had been performed in 3 eyes (20%) with PDR. Before 25GMIVS, intravitreal injection of bevacizumab (IVB) was performed in 4 eyes (27%) with NVG, but after 25GMIVS it was not necessary. There were 9 eyes (60%) with pseudophakia before 25GMIVS.

A summary of the patients' characteristics and post-25GMIVS course is shown in Table 2. PEA, IOL, and 25GMIVS were performed together in 4 eyes (27%), and 25GMIVS was performed by itself in 11 eyes (73%). None of the eyes required suturing of the 25-gauge sclerotomy site at the end of the initial surgery except one (7%) that had undergone vitrectomy before TLE. The mean operative time was 38.3 ± 16.3 minutes. The mean decimal pre-25GMIVS BCVA and final BCVA were 0.05 and 0.3, respectively. Final BCVA in logMAR units was significantly better than the pre-25GMIVS BCVA (*P* = 0.01). Pre-TLE IOP, pre-25GMIVS IOP, and final IOP were 36.0, 11.9, and 15.7 mmHg, respectively. There were significant IOP differences pre-TLE, pre-25GMIVS, and post-25GMIVS (*P* < 0.001), but no significant difference between the pre-25GMIVS IOP and final IOP (*P* = 0.20). There was no difference in the pre-25GMIVS and final number of glaucoma medications (*P* = 0.14). Subconjunctival hemorrhage occurred in 5 eyes (33%); however, in 3 of these eyes, there was no hemorrhage invasion into the filtering bleb. Four eyes (27%) had intraocular pressure >20.0 mmHg after 25GMIVS, and 3 of these eyes needed additional TLE. It should be mentioned that this additional TLE was a technically simple procedure, because 25GMIVS had been performed without any sutures. Vitreoretinal diseases in all 15 eyes with preexisting TLE were successfully treated with 25GMIVS. We achieved success with 25GMIVS in 10 cases (67%) and did not observe surgical complications, such as bacterial endophthalmitis, associated with either TLE or 25GMIVS in any of the cases. The mean follow-up period was 11.5 ± 7.7 months with a range of 6 to 34 months.

## 4. Discussion

We set out to evaluate the feasibility of using 25GMIVS to treat vitreoretinal disease in glaucomatous eyes that had undergone TLE. The most common vitreoretinal disease in eyes with preexisting TLE we treated with this technique was PDR complicated by NVG (in almost 50% of cases). In spite of the relatively high incidence of such a severe disease, mean BCVA improved significantly after 25GMIVS. Additionally, although 3 eyes needed further TLE following our 25GMIVS procedure, the final measurement of IOP was statistically comparable to IOP before 25GMIVS, without an increase in the number of glaucoma medications. Furthermore, because of our use of sutureless 25GMIVS, the additional TLE procedure itself, following 25GMIVS, was not more difficult than a standard TLE procedure.

Our results confirm existing data that, in about one-third of cases, eyes will develop elevated pressure if they undergo vitrectomy after TLE [4]. About 30% of our case series had IOP ≥20.0 mmHg following 25GMIVS, and 3 cases needed additional TLE. In the 3 eyes with types of glaucoma other than NVG, IOP was maintained ≤21 mmHg after 25GMIVS and further TLE was not necessary. In almost 50% of post-TLE eyes we treated with 25GMIVS, however, PDR with NVG was present (Thompson et al; 13% of eyes had PDR) [4]. Our final result for IOP control could thus be considered reasonably successful, given that NVG is known to be generally refractory to conventional TLE with MMC. Specific prognostic factors for surgical failure have been reported to be young age, previous vitrectomy, and, when PDR is present, a fellow eye with NVG [25]. An alternative technique to effectively reduce elevated IOP in eyes with NVG is vitrectomy and complete pan-retinal photocoagulation combined with TLE [26, 27]. Our results, which also support a single existing case report on 25GMIVS for a FAP eye with a filtering bleb, show that 25GMIVS has the potential to become the treatment of choice for vitrectomy in glaucomatous eyes that have already undergone TLE [24]. Additionally, our study now shows that there is no hypotony (IOP < 5.0 mmHg) after 25GMIVS. Thompson et al. reported that IOP outcomes after 20GPPV were rather variable; one-third of eyes in that study developed hypotony, one-third developed elevated pressure, and one-third maintained bleb function [4]. The cause of this discrepancy with our results is unclear, but we speculate that one reason was the high retinal reattachment rate that results from 25GMIVS. The hypotonic eyes from the earlier report included some with persistent retinal detachments following vitrectomy [4]. Additionally, we also believe that the smaller gauge required less infusion of balanced solution during the procedure and had less of a negative intraoperative effect on the ciliary bodies, which have the important role of producing the intraocular aqueous humor.

We speculate that subconjunctival hemorrhage during 25GMIVS affects the preexisting filtering bleb. Many earlier reports have demonstrated the advantages and disadvantages of using autologous blood injection to treat overfiltering or leaking blebs after glaucoma surgery [28–33]. Thus, the sutureless nature of 25GMIVS, which prevents intraoperative subconjunctival hemorrhage from flowing into preexisting bleb, could be a great benefit for the treatment of vitreoretinal disease in eyes with preexisting TLE. We also believe that 25GMIVS can prevent conjunctival adhesion, thereby preserving an existing filtering bleb and clearing the way for additional glaucoma surgery. Subconjunctival hemorrhage after 25GMIVS occurred in about 30% of our cases, with consequent additional glaucoma surgery (about 20%). We thus find it highly advisable to avoid disrupting conjunctival vessels when creating a 25-gauge sclerotomy in glaucomatous eyes that have undergone TLE. The prognostic factors for surgical failure of TLE with MMC in vitrectomized eyes have been reported to be high preoperative IOP and NVG [34]. As stated above, we had 3 NVG patients undergo additional TLE following 25GMIVS, and we were able to achieve a final reduction in IOP in all 3 eyes. We believe that one reason for our success in such severe cases was the conjunctiva's good condition following the sutureless 25GMIVS procedure, in contrast with the earlier report on vitrectomy for NVG, in which a conventional 20GPPV procedure was used. The good condition of the conjunctiva after 25GMIVS might also make it possible to implant recently introduced glaucoma drainage devices, which can aid in the management of complicated glaucoma such as NVG. The need for suturing of a 25-gauge sclerotomy at the end of the 25GMIVS procedure in one vitrectomized eye (patient 5) due to high leakage leads us to believe that vitrectomy can be difficult to perform multiple times without any scleral suturing. In addition, it is difficult to perform IVB in vitrectomized eyes with NVG (patients 5, 12, and 13), because, as has already been demonstrated, injected bevacizumab is quickly washed away from a vitrectomized eye [35]. However, there is one report demonstrating that IVB before TLE might further improve the surgical success rate for NVG in previously vitrectomized eyes [36].

There were limitations to our study, including the retrospective nature of the analysis, a short follow-up period, and a small number of patients. We did not discuss filtering bleb function in detail because our study included eyes with blebs whose function before 25GMIVS was doubtful (these eyes continued to need glaucoma medication after TLE). Furthermore, we did not compare 25GMIVS and 20GPPV. A comparative, prospective study in post-TLE eyes would provide valuable insights into the relative value of these techniques but is impossible due to ethical considerations, making an experimental analysis using an animal model perhaps the most useful approach for such a future study. Nevertheless, as post-TLE eyes that require glaucoma medication for IOP control before vitrectomy are commonly observed clinically and indeed comprised about 50% of the cases in our study, we believe that this is a valuable study of a useful treatment for post-TLE eyes with various retinal conditions. Further investigation is needed to evaluate postoperative visual quality and complications in the late postoperative period before a final determination can be made of the efficacy of this procedure.

In conclusion, 25GMIVS is technique that can feasibly be used to treat vitreoretinal disease in glaucomatous eyes that have undergone TLE. Regardless of a high incidence of PDR with NVG in our case series, we were able to achieve good final results for BCVA and IOP with 25GMIVS, without increasing the number of glaucoma medications. Though there were a few cases that needed additional TLE following 25GMIVS, the additional TLE procedure was not more technically difficult than usual, because of the good condition of the conjunctiva after sutureless 25GMIVS. Our results showed that 25GMIVS was effective in preserving the filtering bleb and the other quadrant conjunctiva in eyes with glaucoma and did not exclude further surgical intervention for this disease.

## Figures and Tables

**Table 1:** Characteristics and pre-25-gauge microincision vitrectomy course of 15 glaucomatous eyes.

Patient no./sex/ age, yrs	Eye	Type of glaucoma	Pre-25GMIVS retinal disease	Period of 25GMIVS after TLE (M)	Site of bleb	Pre-TLE vitrectomy	Pre-25GMIVS IVB	Pre-25GMIVS pseudophakia
1/M/45	L	Trauma	RRD	4	Upper temporal	N	N	Y
2/M/58	L	NVG	BRVO/VH	30	Upper nasal	N	N	N
3/M/62	R	NVG	PDR/VH	48	Upper temporal	N	Y	Y
4/F/60	L	NVG	PDR/TRD	36	Upper nasal	N	Y	N
5/M/68	L	NVG	PDR/VH	10	Upper nasal	Y	Y	Y
6/F/62	L	Uveitis	MH	55	Upper nasal	N	N	Y
7/F/44	R	NVG	PDR/VH/CD	0.5	Upper	N	Y	Y
8/F/83	R	Malignant glaucoma	Malignant glaucoma	1	Upper nasal	N	N	Y
9/F/33	L	Developmental glaucoma	Lens luxation	105	Upper nasal	N	N	N
10/M/52	R	POAG	Endophthalmitis	0.3	Upper temporal	N	N	N
11/M/59	R	POAG	ERM	50	Upper temporal and nasal	N	N	N
12/M/61	R	NVG	PDR/VH	25	Upper temporal	Y	N	Y
13/F/56	R	NVG	PDR	7	Upper temporal	Y	N	Y
14/F/77	L	Malignant glaucoma	Choroidal hemorrhage	1	Upper temporal	N	N	Y
15/M/41	R	NVG	PDR/VH	6	Upper nasal	N	N	N

Mean 57.4		NVG 53%	PDR 47%	25.3		20%	27%	60%

25GMIVS: 25-gauge microincision vitrectomy surgery; TLE: trabeculectomy; IVB: intravitreal injections of bevacizumab; NVG: neovascular glaucoma; TRD: tractional retinal detachment; RRD: rhegmatogenous retinal detachment; BRVO: branch retinal vein occlusion; PDR: proliferative diabetic retinopathy; VH: vitreous hemorrhage; MH: macular hole; ERM: epiretinal membrane; POAG: primary open angle glaucoma; CD: choroidal detachment.

**Table 2:** Characteristics and post-25-gauge microincision vitrectomy course of 15 posttrabeculectomy eyes.

Patient no./ sex/age (y)	Decimal VA course		IOP (mmHg) course			Number of glaucoma medications		25GMIVS			Post-25GMIVS hyposphagma	Post-25GMIVS interventions	Followup (M)	Post-25GMIVS time before failure	Post-25GMIVS success
	Pre-25GMIVS	Final	Pre-TLE IOP	Pre-25GMIVS	Final	Pre-25GMIVS	Final	Combined cataract surgery	Port suturing	Operative time (min)					
1/M/45	1.2	1.2	29	9	9	0	0	N	N	41	N	N	12	—	Y
2/M/58	HM	0.8	35	15	15	0	2	Y	N	17	N	N	6	—	Y
3/M/62	CF	0.01	36	7	19	3	2	N	N	35	N	TLE	9	0.5	N
4/F/60	0.02	0.03	46	21	26	1	4	Y	N	56	Y	N	6	4	N
5/M/68	HM	1.2	37	11	10	0	0	N	Y	41	Y	N	8	—	Y
6/F/62	0.9	1.2	38	7	7	0	0	N	N	22	N	N	6	—	Y
7/F/44	CF	0.2	56	16	19	0	4	N	N	45	Y	TLE	13	8	N
8/F/83	1	1.2	30	30	13	3	0	N	N	25	N	N	12	—	Y
9/F/33	0.15	0.9	32	12	20	0	0	Y	N	74	N	N	9	—	Y
10/M/52	HM	0.7	16	7	16	3	4	N	N	26	Y	N	34	—	Y
11/M/59	0.6	0.6	19	10	15	0	2	Y	N	26	N	N	6	—	Y
12/M/61	HM	0.6	30	9	16	1	2	N	N	22	N	N	23	—	Y
13/F/56	HM	NLP	49	9	18	3	3	N	N	35	N	N	13	13	N
14/F/77	HM	0.08	32	10	17	3	3	N	N	59	Y	N	7	—	Y
15/M/41	0.03	HM	55	6	16	1	2	N	N	50	N	TLE	8	1	N

Mean 57.4	0.05	0.3	36.0 mmHg	11.9	15.7	1.2	1.9	27%	7%	38.3	33%	20%	11.5		67%

25GMIVS: 25-gauge microincision vitrectomy surgery; IOP: intraocular pressure; VA: visual acuity; IOP: intraocular pressure; TLE: trabeculectomy; HM: hand movement; CF: counting fingers; NLP: no light perception.

*P* < 0.01; Wilcoxon signed-ranks test; pre-25GMIVS VA versus final VA.

*P* < 0.001; Friedman test for 3 groups: pre-TLE IOP, pre-25GMIVS IOP and final IOP.

*P* = 0.50; Scheffe's test; pre-25GMIVS IOP versus final IOP.

*P* = 0.67; Wilcoxon signed-ranks test; pre-25GMIVS number of glaucoma medications versus final number of glaucoma medications.
